# Microbial biodiversity contributes to soil carbon release: a case study on fire disturbed boreal forests

**DOI:** 10.1093/femsec/fiac074

**Published:** 2022-06-24

**Authors:** Xuan Zhou, Hui Sun, Jussi Heinonsalo, Jukka Pumpanen, Frank Berninger

**Affiliations:** Department of Environmental and Biological Sciences, University of Eastern Finland, Joensuu campus, 80101 Joensuu, Finland; Collaborative Innovation Center of Sustainable Forestry in China, College of Forestry, Nanjing Forestry University, 210037 Nanjing, China; Department of Forest Sciences, University of Helsinki, 00014 Helsinki, Finland; Department of Environmental and Biological Sciences, University of Eastern Finland, Joensuu campus, 80101 Joensuu, Finland; Department of Environmental and Biological Sciences, University of Eastern Finland, Joensuu campus, 80101 Joensuu, Finland

**Keywords:** bacterial diversity, fungal diversity, microbial community composition, microbial functional genes, soil carbon emission, structural equation modelling

## Abstract

Microbial communities often possess enormous diversity, raising questions about whether this diversity drives ecosystem functioning, especially the influence of diversity on soil decomposition and respiration. Although functional redundancy is widely observed in soil microorganisms, evidence that species occupy distinct metabolic niches has also emerged. In this paper, we found that apart from the environmental variables, increases in microbial diversity, notably bacterial diversity, lead to an increase in soil C emissions. This was demonstrated using structural equation modelling (SEM), linking soil respiration with naturally differing levels of soil physio-chemical properties, vegetation coverage, and microbial diversity after fire disturbance. Our SEMs also revealed that models including bacterial diversity explained more variation of soil CO_2_ emissions (about 45%) than fungal diversity (about 38%). A possible explanation of this discrepancy is that fungi are more multifunctional than bacteria and, therefore, an increase in fungal diversity does not necessarily change soil respiration. Further analysis on functional gene structure suggested that bacterial and fungal diversities mainly explain the potential decomposition of recalcitrant C compare with that of labile C. Overall, by incorporating microbial diversity and the environmental variables, the predictive power of models on soil C emission was significantly improved, indicating microbial diversity is crucial for predicting ecosystem functions.

## Teaser

Microbial biodiversity plays the dominant role in soil carbon emissions in fire-disturbed boreal forests.

## Introduction

Soil carbon (C) emissions in boreal forests, especially in permafrost, have received great attention due to the thick layer of soil organic matter, acting as a potential source for atmospheric carbon dioxide (CO_2_). Increasing fire frequency in boreal forest due to climate change (Randerson et al.^2006^) has altered the C input from the vegetation and permafrost stability in boreal ecosystem (Neff, Harden and Gleixner 2005). Boreal wildfires indirectly result in the thawing of near-surface permafrost and a deepening of the active layer (Johnstone et al. 2010). In a long-term scale, fire-induced disturbance can have large direct and indirect effects on soil microbial community due to the shifts in environmental conditions.

Soil microorganisms account for a large part of the biodiversity on Earth, with over a billion microbial cells and about 30–50 thousand taxa per gram of soil (Roesch et al. 2007). Athough biodiversity of other organisms has long been recognized to be closely associated with the functions and the stability of ecosystems (Balvanera et al. 2006, Reich et al. 2012), the influence of microbial diversity on ecosystem processes still remains unresolved. This is mainly because of the functional redundancy within the microbial community, which denotes that the shifts in microbial community composition do not necessarily change ecosystem processes since different species often share similar functions in the ecosystem (Louca et al. 2018). Thus, most terrestrial ecosystem models assume that the decomposition rate of soil organic matter (SOM) is mainly determined by abiotic conditions and is deemed to be invariant with shifts in the microbial communities (Louca et al. 2018).

Evidence for functional redundancy, however, is mixed. Increasing evidence about the relationship between phylogenetic diversity and microbial functions (Wagg et al. 2014, Delgado-Baquerizo et al. 2016, Maron et al. 2018) and soil respiration (Tilman, Wedin and Knops 1996, Zavaleta et al. 2010, Schimel and Schaeffer 2012) is emerging, indicating microbial community structure and diversity affects the functions of the soil microbiome. Given soils contain a highly diverse mixture of organic compounds, microbial taxa ought to occupy distinct niches to compete for limited carbon (C) resources (Zhou et al. 2002). The presence of a diverse microbial community therefore enhances its metabolic potential to decompose a larger spectrum of C substrates and thus increase C release. For instance, oxidations of xylose-C and vanillyl-lignin were increased when microbial Shannon diversity was high, revealing that microbial diversity alters the decomposition of complex C substrates (Baumann et al. 2013). However, opposing results have also been found that a highly diverse community results in reduced litter decomposition rates, as demonstrated for wood decomposing fungi (Boddy 2000).

Microorganisms mineralize and assimilate different types of C resources mainly depending on the complexity of these materials (Kramer et al. 2016). Schimel and Schaeffer (2012) classified microbial functions into ‘broad’ functions and ‘narrow’ functions. Resources that are readily decomposed (broad function) possess many different species to decompose, while the ones that are more recalcitrant (narrow functions) require different species possessing complementary enzymes to be adequately decomposed. Thus, broad functions are generally considered to be redundant, while narrow functions that deal with more complex pathways are restricted to only a few phylogenetic groups. Many studies using manipulated microbial communities showed that the soil respiration rates and C degradation are, indeed, affected by the shifts in microbial diversity (Strickland et al. 2009, McGuire and Treseder 2010, Glassman et al. 2018, Liu et al. 2018). However, other studies also found no effect of microbial diversity on C decomposition (Griffiths et al. 2001, Wertz et al. 2006). Hence, whether microbial diversity drives soil respiration, especially its interactions with climate, soil properties and plant features have not yet been addressed comprehensively.

Fungi and bacteria play fundamental roles in decomposing SOM (Swift, Heal and Anderson 1979). Fungi are known to be the major contributors to a wide range of other organic compounds (Baldrian et al. 2011), particularly recalcitrant organic matters, such as lignin and cellulose (De Boer, Kowalchuk and Van Veen 2006, Dighton 2018). Monocultures of decomposer fungi have shown that different species have their own resource niche (Cox, Wilkinson and Anderson 2001, Deacon et al. 2006, Boberg, Ihrmark and Lindahl 2011). In studies involving two to five species of fungi, decomposition rates significantly exceeded the ones in monocultures (Treton, Chauvet and Charcosset 2004, Tiunov and Scheu 2005). However, the C decomposition rate seems to be saturated at low fungal species richness, indicating fungi are functionally redundant (Nielsen et al. 2011). Furthermore, it is also found that a considerable overlap in the ability to decompose certain C compounds between different fungal clades (Deacon et al. 2006, Talbot et al. 2014). In contrast, different bacterial clades seem to be more specific to decompose certain types of C compounds. For instance, an experimental microcosm study on bacterial diversity found a strong correlation between microbial respiration and species richness when richness increased from 1 to 72 taxa (Bell et al. 2005). An inoculation experiment also showed that bacterial communities change their composition to acclimate to new environments while fungal communities do not necessarily change to adapt to a new habitat (Glassman et al. 2018). All these studies suggest that bacteria are less functionally redundant than fungi and the shifts in bacterial diversity can potentially have a larger impact on soil respiration than shifts in fungal diversity.

To better address the complex networks in the ecosystem, we use structural equation modelling (SEM), a form of path analysis that resolves complex relationships among interrelated variables (Lefcheck 2016). Instead of the direct bivariate relationships, SEM considers the mediators within a series of causal networks to advance our understanding of the ecosystem. Here, we use data from four successional stages of boreal forests after a wildfire in Canada, which consist of soil properties, vegetation coverages, microbial biomasses and diversities, and soil CO_2_ fluxes. Based on the intermediate disturbance hypothesis (Connell 1978), high species diversity appears at an intermediate-level disturbance, whereas the environment lacking disturbance or under severe disturbance yields low biodiversity. This fire choronosequence experiment forms a natural gradient of soil diversity recovered from a fire disturbance, so as a gradient of soil properties and plant features. We compare several models to test hypotheses on the relationships between the environment, the microbiome, and soil respiration.

Here we posit that microbial diversity can potentially contribute to large portions of the variation in soil respiration which cannot be explained by the other biotic and abiotic variables. We also hypothesize that shifts in bacterial diversity affect soil respiration more than shifts in fungal diversity. In addition, given microbial diversity reflects the shifts in microbial communities, the changing community composition will be discussed for a deeper understanding of which taxa groups contribute to the potential functions of decomposition. Estimating the biodiversity of microbial communities is challenging due to the huge number of organisms in microbial communities compared with those in plant or animal communities (Whitman, Coleman and Wiebe 1998). It therefore leads to severe sampling bias (i.e. sequencing depth). Shannon diversity index, which considers the number of phylotypes in one habitat (richness) and their relative abundance (evenness), has been proved to be less sensitive to sampling bias (Haegeman et al. 2013). Thus, herein, we use the Shannon diversity index to predict soil respiration rate.

## Materials and methods

### Site description and data collection

We used combined data from a fire chronosequence study presented in Köster et al. (2017), Aaltonen et al. (2019), Zhou et al. (2019), and Zhou et al. (2020). Data availability is presented in Table 1. The fire chronosequence study collected four boreal forest areas in the Yukon and the Northwest Territories (Canada, 66°22′ N–67°26′ N, 136°43′ W–133°45′ W) that were burned 3, 25, 46 years ago, and a control area that was absent from fire for at least 100 years. Postfire ages of the burned areas were determined based on Canadian government GIS data (GeoYukon 2011), and the age of the control was determined by taking increment cores from the largest tree in the sampling plot. The mean annual temperature of these areas is -8.8 °C. The areas are dominated by *Picea marina* (Mill.) and *Picea glauca* Voss species (Köster et al. 2017). In this paper, we use the ages from the last fire (Forest age) until the sampling year (July 2015 during the growing season) as a continuous variable (3, 25, 46, 100 years). Areas were coded as Fire_3_, Fire_25_, Fire_46_, and Fire_100_, respectively.

**Table 1.** The 25 percentage and 75 percentage quantiles of the predictors involved in the structural equation modelling and the correlation test.

Variables	Abbreviation	Model	Depth	Fire_3_	Fire_25_	Fire_46_	Fire_100_	Reference
Micro-climate (latent variable)	Temp (^o^C)	SEM_1-4_	5 cm	5.8–8.4	7.1–7.1	6.8–11.3	6.1–8.0	Köster et al. (2017)
			10 cm	4.4–5.9	4.5–6.1	4.1–6.1	2.0–3.3	
	Activ.layer.depth (m)			0.78–1.48	0.53–1.32	0.53–0.80	0.26–0.32	
	AvMoi (%)	SEM_1-4_	5 cm	33.1–40.7	30.0–42.0	41.6–51.1	45.7–66.2	
			10 cm	33.1–43.9	38.5–50.7	41.4–51.1	38.7–64.6	
Soil organic matter	SOM (g g^–1^)	SEM_1-4_	5 cm	0.16–0.91	0.13–0.47	0.13–0.77	0.84–1.00	
			10 cm	0.04–0.09	0.07–0.23	0.11–0.36	0.50–0.90	
Root content	Roots (g g^–1^)	SEM_1-4_	5 cm	0.02–0.07	0.05–0.11	0.16–0.37	0.10–0.13	Aaltonen et al. (2019)
			10 cm	0.002–0.003	0.02–0.05	0.06–0.11	0.02–0.07	
Dissolved organic C	DOC (mg g^–1^)	SEM_1-4_	5 cm	0.88–3.26	0.66–2.11	1.73–6.06	4.78–15.15	Zhou et al. (2019)
			10 cm	0.26–0.34	0.33–1.08	0.52–0.91	1.16–2.64	
Microbial biomass	MB (mg g^–1^)	SEM_1-4_	5 cm	0.8–5.3	1.26–4.8	1.0–8.8	5.7–14.5	
			10 cm	0.09–0.15	0.45–3–62	0.67–1.87	2.69–4.10	
Microbial diversity (latent variable)	H_Bact_	SEM_1,3_	5 cm	5.22–5.46	5.38–5.48	5.50–5.78	4.94–5.40	Zhou et al. (2020)
			10 cm	5.45–5.62	5.13–5.57	5.73–5.85	5.17–5.75	
	H_Fungi_	SEM_1,4_	5 cm	3.08–3.67	2.66–3.07	3.09–3.23	2.61–3.28	
			10 cm	2.72–3.42	2.74–3.18	3.29–3.59	2.56–3.39	
Soil repsiration	CO_2_ (mg CO_2_ s^–1^m^–2^)	SEM1-4		0.10–0.19	0.15–0.39	0.40–0.54	0.22–0.39	Köster et al.(2017)
Microbial heterotrphic respiration	CO_2_ emission (incubation at 19 °C) (mg CO_2_ gC^–1^h^–1^)	Correlat-ion test	5 cm	0.09–0.18	0.08–0.23	0.05–0.12	0.17–0.75	Aaltonen et al. (2019)
			10 cm	0.03–0.09	0.03–0.11	0.003–0.02	0.09–0.15	
			30 cm	0.016–0.021	0.016–0.047	0.001–0.002	0.025–0.029	

At each fire area, we established three sampling lines that were at least 200-m apart, and within each line, three plots in size of 400 m^2^ were established. At each plot, soil samples were taken from three layers down to 30 cm (5, 10, and 30 cm). Briefly, soils were subsampled from homogenized soils taken from a cylinder that was horizontally inserted at each soil depth. In total, 107 soil samples [4 forest areas × 3 lines × 3 plots × 3 layers, with one sample missing] were available for analysis.

In the current study, two types of soil respiration were measured. Soil CO_2_ emission from the soil surface in the field was measured using the static chamber method (Köster et al. 2017). This type of CO_2_ emission consists of respiration from soil microbes (heterotrophic respiration) and plant roots (autotrophic respiration). This data was used to build the models since it reflects the actual respiration from the soil and is able to detect the causal relationships between the complex environmental variables in nature. Another type of soil respiration, i.e. heterotrophic respiration, was estimated using incubation experiments. Prior to the incubation, roots were removed from the soils. The respiration of the soils was measured during the incubation at 19°C for 24 h. The data and the method are described in more detail in Aaltonen et al. (2019). The CO_2_ fluxes estimated by incubation represent only heterotrophic microbial respiration and do not include root respiration. However, it is worth noting that microbial respiration measured here only reflects the CO_2_ fluxes under identical conditions (same temperature and moisture), rather than the real heterotrophic respiration would have been in the field.

At each plot and soil layer, we also measured soil temperature and moisture, the active layer depth of the permafrost, SOM, soil dissoved organic C (DOC), microbial biomass, bacterial and fungal community composition, and other auxiliary variables, as described previously (Köster et al. 2017, Zhou et al. 2019, 2020). Briefly, DOC content was measured using the total organic C analyzer (Shimadzu TOC-V CPH, Shimadzu Corp., Kyoto, Japan) from soil K_2_SO_4_ (0.5 M) extracts. The soil root biomass was measured by weighing the roots taken from the soil samples. SOM was estimated by the weight loss after burning under 550 °C in the oven. Microbial biomass was estimated using the chloroform fumigation method (Hedley and Stewart 1982, Zhou et al. 2019)

### Microbial biodiversity and microbial functional genes

The bacterial and fungal community composition were detected by primer sets of f341/r785 (bacteria) (Klindworth et al. 2013) and gITS7/ITS4 (fungi) (Ihrmark et al. 2012), respectively, using Illumina MiSeq sequencing at the Institute of Biotechnology, University of Helsinki. The raw sequencing data of the bacterial community and fungal community are available in the NCBI (National Centre for Biotechnology Information) database under Bioproject number PRJNA514982 and PRJNA780219, respectively. Details of the downstream analysis of the sequencing data were described in Zhou et al. (2020). Briefly, sequence data were aligned to operational taxonomic units (OTUs) using mothur pipeline (version 1.39; Schloss et al. 2009). To avoid the influence of rare species, we removed rare OTUs that presented less than 5% of the total sample size (n = 107). This process has little effect on the Shannon diversity index since the Shannon index takes into account both richness and relative abundance (evenness) of the phylotypes in one community, which is less sensitive to the sampling size or sequencing depth (Haegeman et al. 2013). The Shannon diversity index was calculated using the natural logarithm.

Microbial potential functions were detected using GeoChip 5.0 K (Glomics Inc.) with combined DNA samples from three replicate plots in each line at 5 and 30 cm layers. Therefore, 24 samples were available for functional gene profiles. The detailed information about processing the samples for GeoChip 5.0 K was described in Zhou et al. (2020). Briefly, The genomic DNA was mixed with random primers and then labelled with a fluorescent labelling master mix. The labelled genomic DNA was further purified, hybridised and scanned in the GeoChip 5.0 K microarray (Van Nostrand et al. 2016). High-quality fluorescent intensity was obtained from Glomics Inc. and then normalised by dividing each gene frequency by the mean of the total frequency of the gene category to which that gene belongs (Van Nostrand et al. 2016). The method for detecting functional genes was DNA-based, and thus the caveat of this approach is that the functional genes reflect only the potential functions, rather than the active microbial functions.

### Description of SEM model

We posit that disturbance of wildfires affects vegetation, soil conditions, and the micro-climate, which change the structure and functionality of microorganisms and therefore soil respiration. To simultaneously discover each of these pathways, SEM was used by accounting for correlations between and through multiple response variables (Bollen 1969). By doing so, we can separate the causal relationships between many variables included in the model, and partition the direct effects into several indirect ones, revealing intact networks in the ecosystem. All SEM models were conducted using *lavaan* package in R (Rosseel 2012). The variables that violated the SEM assumption of multivariate normality (Shapiro-Wilk's test, *P* < 0.05) were log-transformed. The log-transformed variables were listed in Table S1 and they all correspond with normality after transformation.

**Figure 1.**
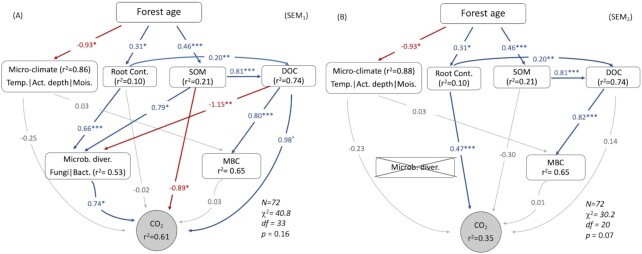
Prediction of soil CO_2_ fluxes along forest succession after a fire using paths with microbial diversity **(A)** and without microbial diversity**(B)**. Blue solid lines represent positive correlations and red lines are negative correlations, whereas the grey lines show the paths that are not significant. The standardized coefficients for indicating the strength of the effects are presented on the significant paths with blue (positive) and red (negative) numbers. Significant levels are as follows: *P < 0.05; **P < 0.01; ***P < 0.001. The r^2^ [ = (1-residual variance)/observed variance] indicates the variance of the variable explained by the direct and indirect pathways pointing towards it.

To simulate C emissions from the ecosystems, CO_2_ fluxes measured at soil surface (5 and 10 cm layers) in the study areas were used in SEMs as soil respiration, which consists of both autotroph and heterotroph respiration. Along with the recovery time after a fire, plant roots and microorganisms gradually enter the edaphic ecosystem that contributes to soil respiration. Thus, the direct effects of root biomass, microbial characteristics (microbial biomass and microbial diversity), as well as SOM, and DOC on soil respiration were included in our models. In addition, root biomass, SOM, DOC, and micro-climate were involved as indirect variables to explain soil respiration through microbial diversity and microbial biomass (Fig. 1). Micro-climate that consists of soil temperature, active layer depth, and moisture was set as a latent variable. Another latent variable is microbial diversity (Fig. 1A), which consists of bacterial and fungal Shannon indices.

**Figure 2.**
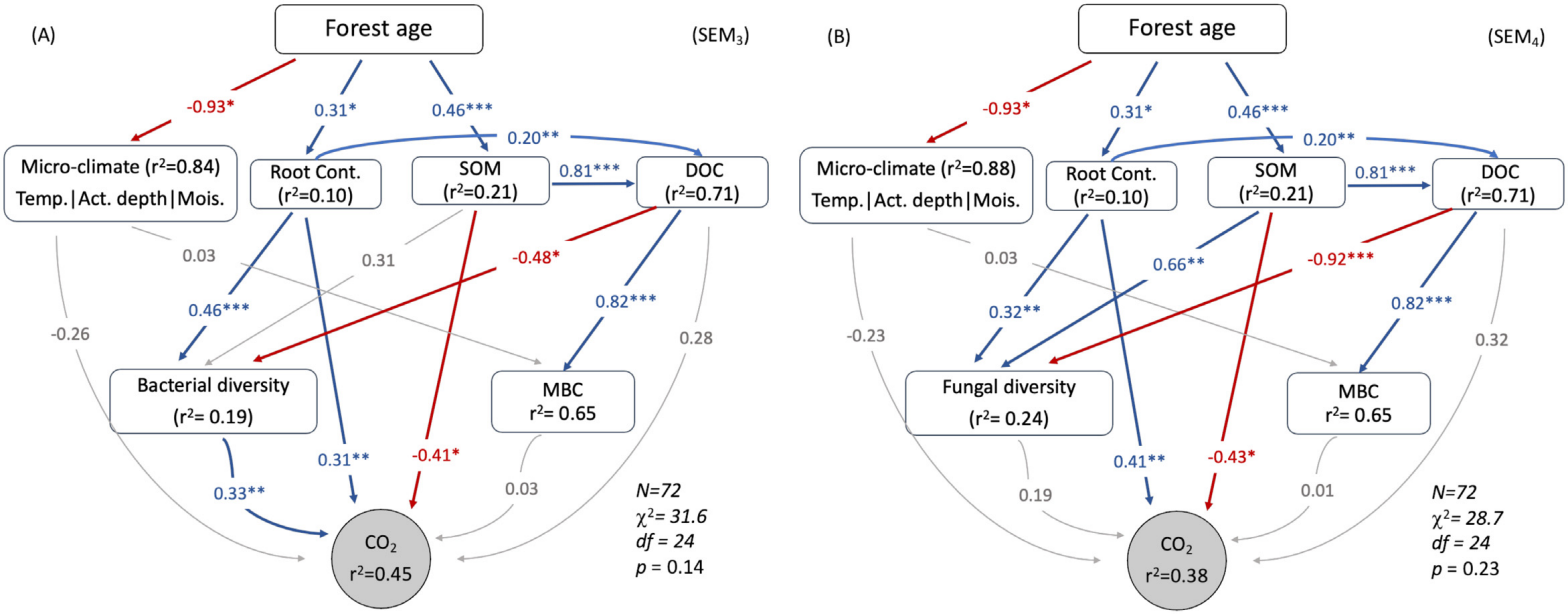
Comparison between the effect of bacterial diversity **(A)** and fungal diversity **(B)** on soil CO_2_ fluxes along forest succession after a fire. Blue solid lines represent positive correlations and red lines are negative correlations, whereas the dashed grey lines show the paths that are not significant. The standardized coefficients for indicating the strength of the effects are presented on the significant paths with blue (positive) and red (negative) numbers. Significant levels are as follows: *P < 0.05; **P < 0.01; ***P < 0.001. The *r^2^* [ = (1-residual variance)/observed variance] indicates the variance of the variable explained by the direct and indirect pathways pointing towards it.

To test our hypothesis of whether microbial diversity contributes to soil respiration, two SEM models were established. We compared the model including the paths with microbial diversity (SEM_1_; Fig. 1A) and the model without those paths (SEM_2_; Fig. 1B). We further compare the model with bacterial diversity and the other with fungal diversity: one replaces the path of microbial diversity in SEM_1_ with bacterial diversity (SEM_3_; Fig. 2A) and the other replaces the same path with fungal diversity (SEM_4_; Fig. 2B). SEM model fits were tested using a Satorra-Bentler corrected likelihood ratio χ^2^ test, where models with 0 < χ^2^/df ≤ 2 and *P* > 0.05 are considered as good fit (Trivedi et al. 2016). In our analysis, we did not attempt to build a ‘parsimonious’ model by removing insignificant paths from the model but retained all plausible paths from the *priori* models.

### Other statistical analyses

We further tested the effect of microbial diversity and community composition on the potential decomposition of soil C compounds using the functional gene profiles that were determined by GeoChip 5.0 K. Genes involved in C degradation were categorized by their encoded enzymes, each of which decomposes one of the C compounds. The main C compounds were starch, lactose, hemicellulose, terpenes, chitin, cellulose, lignin, and tannins. Despite the ambiguity of the definition of recalcitrance (Kleber and Kleber 2010), hemicellulose, which possesses a heteropolymeric structure composing of various sugar monomers, is considered as recalcitrant organic carbon in this paper. Thus, starch and lactose were considered labile C and hemicellulose, chitin, cellulose, lignin, and tannins are considered recalcitrant C. Multivariate analyses were applied to test whether microbial diversity and community composition explain the variations of functional genes. Briefly, a principal component analysis (PCA) using data of functional gene frequencies was first conducted, and bacterial diversity and fungal diversity were later fitted on the PCA ordination. The same method was also used for fitting bacterial community (at phylum level) and fungal community (at class level) to the PCA ordination. In addition, to test why microbial biomass fail to explain soil respiration measured in the field, we compared two multiple linear regressions using respiration measured from the field and the one from incubation experiments.

All these statistical analyses were conducted in R (version 4.0.3), and the involved packages are *vegan* (Oksanen et al. 2018), *dplyr* (Bunn 2010), and *corrplot* (Taiyun Wei 2021).

## Results

### Bacterial and fungal diversities

A total of 11135 bacterial and 7095 fungal OTUs were collected in the four forest areas (Zhou et al. 2020). The dominant taxa were shown in Fig. S1 and the pattern of bacterial and fungal community compositions (at OTU level) across four areas were shown in Fig. S2. The total abundance of bacterial OTUs in 107 samples ranges from 6 to 169 130 counts and that of fungal OTUs ranges from 1 to 189 677 counts. To avoid the effects of rare species on the community diversities, top 95% of the global abundance of bacteria and fungi, consists of 3404 bacterial OTUs (ranging from 60 to 169 130 counts) and 808 fungal OTUs (ranging from 370 to 189 677 counts), respectively, were retained for further analysis. The reason for sifting rare OTUs is that (i) those OTUs are more likely contain random and problematic OTUs due to sequencing error; (ii) they contribute little to the ecosystem functions; (iii) removing them did not change the pattern of the Shannon diversity calculated by unpruned OTUs (Fig. S3). The bacterial diversity, estimated with the Shannon index ranged from 3.67 to 6.14, and the fungal diversity ranged from 1.61 to 4.69 (Table 1). The lowest diversity appeared in Fire_100_ and the highest diversity appeared in Fire_46_.

### Contributions of microbial diversity and environmental variables to soil respiration

Our results also showed that soil CO_2_ fluxes increased with the recovery time after a fire, through the increase in root biomass, soil dissolved organic C (DOC) and microbial diversity (Fig. 1A). Compared with SEM_1_ with microbial diversity, SEM_2_ without microbial diversity explained 26% less variation in soil respiration (Fig. 1): SEM_1_ (r^2^ = 0.61; Fig. 1A) which includes microbial diversity explained about 61% of the variations in the soil CO_2_ fluxes, while SEM_2_ explained 35% (Fig. 1B). Furthermore, the model fit of SEM_1_ was better than that of SEM_2_: the covariance matrices of SEM_1_ were undifferentiated from the observed covariance matrices (χ^2^ = 41, df = 33, *p* = 0.16), in other words, the modelled results represent the observed data. However, the covariance matrices predicted by SEM_2_ did not strongly represent the observed data (Fig. 1B; χ^2^ = 30.2, df = 20, *p* = 0.07).

Overall, 61% of the variation in CO_2_ emission was explained by the direct and indirect paths in SEM_1_. Although root biomass has no direct effect on CO_2_ emission, it significantly increased microbial diversity which positively correlated with CO_2_ emission. About 53% (r^2^ = 0.53) of the microbial diversity in the SEM_1_ was explained by root biomass, SOM, and DOC content (Fig. 1A). In addition to root biomass, SOM and DOC, which increased along with the recovery time after the fire, had both direct and indirect effects on soil CO_2_ emissions (Fig. 1A). Surprisingly, microbial biomass did not explain much of CO_2_ emission.

By removing the microbial diversity from the model, the variance of CO_2_ emission explained was dropped from 61% (Fig. 1A) to 35% (Fig. 1B). Root biomass became the dominant variable explaining CO_2_ emission, and the effects of SOM and DOC became insignificant. By comparing SEM_1_ and SEM_2_, it is obvious that microbial diversity acted as a crucial mediator between root biomass, SOM, and DOC → CO_2_ paths.

### Comparison between contributions of bacterial and fungal diversity to soil respiration

Another two SEM models were established to test whether bacterial diversity (SEM_3_) or fungal diversity (SEM_4_) contributes to the soil CO_2_ emission (Fig. 2A and B). SEM_3_ with bacterial diversity (r^2^ = 0.45; Fig. 2A) explained higher proportion of the variation in CO_2_ fluxes than SEM_4_ with fungal diversity (SEM_4_, r^2^ = 0.38; Fig. 2B). In addition, the model with only fungal diversity (SEM_4_) explained almost the same amount of variation in CO_2_ fluxes as that explained by SEM_2_ (r^2^ = 0.35; Fig. 1B) where microbial diversity was excluded.

Soil CO_2_ emission was directly correlated with bacterial diversity, root biomass, and SOM, and was also indirectly correlated with root biomass and SOM through bacterial diversity (Fig. 2A). About 19% of the variation in bacterial diversity was explained by root biomass (*P* < 0.05), DOC (*P* < 0.05), and SOM (statistically insignificant).

The direct and indirect effects of fungal diversity, root biomass, SOM, and DOC explained about 38% of soil CO_2_ emissions (Fig. 2B). Although the relationship between fungal diversity and CO_2_ emission was insignificant, it alone explained CO_2_ by about 3%. About 24% of fungal diversity was explained by root biomass, SOM, and DOC (*P* < 0.05; Fig. 2B).

The results of SEM paths between forest age → soil properties, soil properties → microbial biomass → CO_2_ fluxes of all SEMs were relatively similar. Forest age was negatively correlated with soil micro-climate (consists of soil temperature, active layer depth, and soil moisture) and positively correlated with soil root biomass and SOM (*P* < 0.05; Figs. 1 and 2). Soil DOC was positively correlated with root biomass and SOM (*P* < 0.05), which explained 71% of DOC's variation (Figs. 1 and 2). Soil microbial biomass was positively correlated with DOC content, and 65% of it was explained by DOC (*P* < 0.001) and micro-climate.

### Effects of microbial diversity on potential functions of C decomposition

**Figure 3.**
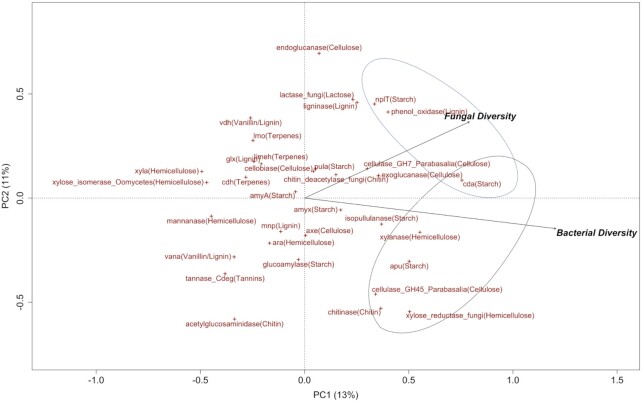
Principal component analysis (PCA) diagram to determine the contribution of bacterial diversity and fungal diversity on the frequencies of genes coding for different carbon compounds. Gene groups within the two circles represent their positive correlations with bacterial diversity and fungal diversity, respectively.

The PCA results showed that bacterial and fungal diversities explained more variation along the PC1 (Fig. 3). Bacterial diversity positively correlated with gene groups of xylanase and xylose reductase (decomposing hemicellulose), cellulase (cellulose), chitinase (chitin), apu and cda (starch), whilst negatively correlated with xyla and xylose isomerase (hemicellulose) (Fig. 3). Fungal diversity has positive correlations with cda and nplT (starch), and phenol oxidase (lignin), whereas negatively correlated with mannanase (hemicellulose), vana (vanillin/lignin), tannase (tannins), and acetylglucosaminidase (chitin) (Fig. 3).

### Contribution of microorganisms on soil respiration

Soil respiration consists of two main resources, that are microbial respiration and root respiration. To predict the portion that contributes to microbial respiration, an incubation experiment of soil microorganisms at 19°C was conducted. The result showed that microbial biomass was significantly correlated with microbial respiration, but not with bacterial and fungal Shannon diversities (Table 2). In contrast, soil CO_2_ emission that was measured directly from the field was explained little by soil microbial biomass, whilst it was positively correlated with bacterial Shannon index (Table 2).

**Table 2.** Parameters of multiple linear models explaining respiration measured from incubation experiment (microbial respiration) and from the field (CO_2_ emission).

Formulas	Variables	Coefficients	*p* values
Microbial respiration (at 19 °C)			
∼ MBC + H_Bact_ + H_Fungi_	MBC	9.74	< 0.01
R^2^ = 0.31; *P* < 0.01	H_Bact_	–	n.s.
	H_Fungi_	–	n.s.
CO_2_ emission			
∼ MBC + H_Bact_ + H_Fungi_	MBC	–	n.s.
R ^2^ = 0.16; *P* < 0.01	H_Bact_	0.21	< 0.01
	H_Fungi_	–	n.s.

### Shifts in microbial taxa groups on potential degradation of different C compounds

**Figure 4.**
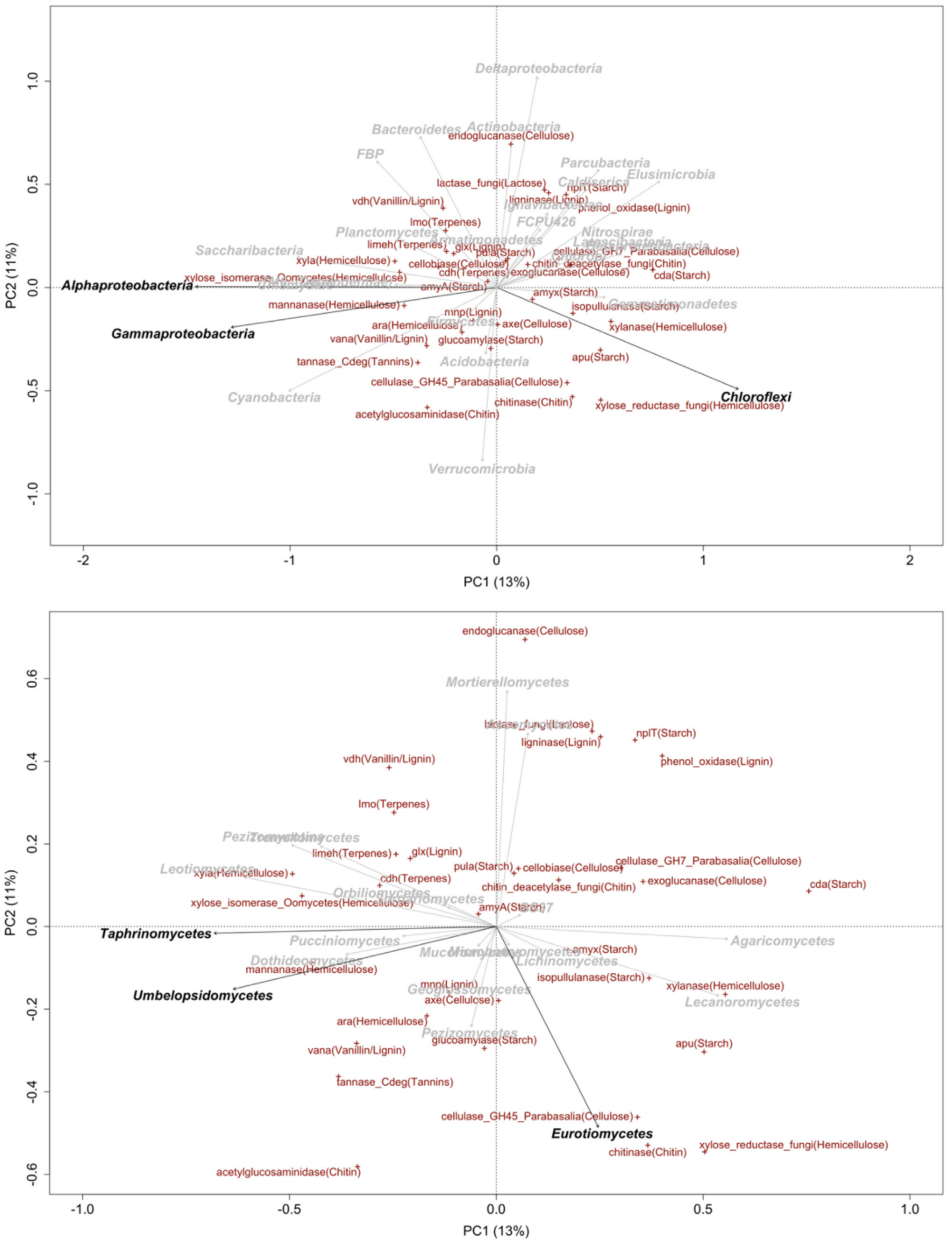
Principal component analysis (PCA) plots to show the ordination of functional genes along the first two axes and their correlation with **(A)** bacterial community (at phylum level) and **(B)** fungal community (at class level). Taxa that significantly (*P* < 0.05) correlated with the first two axes are highlighted in black.

Given biodiversity reflects microbial community composition, it is obliged to examine the effect of different microbial taxa on the decomposition of organic C compounds. Thus, PCA analyses of functional gene frequencies with fitted bacterial taxa (at phylum level; Fig. 4A) and fungal taxa (at class level; Fig. 4B) were conducted. The results showed that Alphaproteobacteria and Gammaproteobacteria were correlated with xylose isomerase, xyla, and mannanase coding for hemicellulose decomposition, and negatively correlated with cda (starch), cellulase, and exoglucanase (cellulose). Chloroflexi was positively correlated with cellulase (cellulose) and xylose reductase (hemicellulose).

Fungal taxa Taphrinomycetes and Umbelopsidomycetes were positively correlated with xylose isomerase, xyla, and mannanase (hemicellulose), and negatively correlated with cda (starch), cellulase and exoglucanase (cellulose). Eurotiomycetes positively correlated with chitinase (chitin), cellulase (cellulose), and xylose reductase (hemicellulose), while negatively correlated with vdh (vanillin/lignin) and imo (terpenes).

## Discussion

Our work demonstrates that higher microbial diversity does, in fire-disturbed forest ecosystems, increase soil respiration. Our results are, thus in conflict with the common implicit assumption of functional redundancy of the soil microbiome (Louca et al. 2018) and support that microbial diversity plays an important role in ecosystem functioning. The model incorporating microbial diversity explains more variation of the soil respiration than the alternative model excluding those paths. Although both bacterial and fungal diversities explain soil respiration to a certain amount, bacterial diversity improves model performance more than fungal diversity. By removing microbial diversity from the model, the proportion of explained variance of soil CO_2_ emissions almost halved (61% to 35%; Fig. 1). Furthermore, potential functions explained by microbial diversity were mainly related to the decomposition of recalcitrant C, indicating that microbial diversity alters narrow functions. Therefore, our current study shows opposing viewpoints from functional redundancy, demonstrating that the changing microbial diversity, reflecting the shifts in microbial community composition, alters the ecosystem functionality of C degradation.

Our SEM results showed that microbial diversity (mainly bacterial diversity) conjointly with environmental factors played a key role in soil CO_2_ emissions. By removing microbial diversity from the model, the model fit and the proportion of explained variance of the soil CO_2_ fluxes dropped (Fig. 1). This implies that increases in soil microbial species richness and abundance do affect ecosystem functions. A community with higher diversity has a higher chance of introducing more taxa groups with diverse functions. This is in accordance with our previous study that bacterial community structure explains about 33% of the bacterial potential functions (Zhou et al. 2020). Previous studies that control microbial diversity using a dilution method found that the reduction in microbial diversity comes along with reductions in several metabolic rates (Salonius 1981, Philippot et al. 2013). An empirical study on grassland soil also found that soil biodiversity positively correlated with decomposition (Wagg et al. 2014). However, it has also been observed that soil respiration remains unchanged with microbial diversity (Griffiths et al. 2001, Wertz et al. 2006). Despite the contradictory results from these studies, they shared a similar dilution method to control microbial diversity, which removes certain species to reduce the diversity. In contrast, the current study estimates the community diversity formed by fire disturbances. The changing diversity can be caused by myriad combinations of different species. In this way, a community with low diversity does not necessarily mean a reduction of certain species from the one with high diversity but may be replaced by another species that are more dominant in this specific environment. Thus, soil respiration measured in the current study can reflect the natural condition.

Changes in fungal diversity contribute less to C emissions compared with bacterial diversity (Fig. 2). A previous study also concluded that shifts in fungal community composition do not necessarily influence their decomposition rate (Van der Wal et al. 2013). This makes fungal communities more functionally redundant. For instance, previous studies address that many metabolic activities of saprophytic soil fungal species are highly overlapping (Deacon et al. 2006, Talbot et al. 2014). In addition, many studies note that fungal decomposition rate and metabolic activity are saturated at rather a low species diversity (Setälä and McLean 2004, Gessner et al. 2010, Nielsen et al. 2011). In addition, the shifts in fungal community composition do not necessarily influence the decomposition processes of the ecosystems (Van der Wal et al. 2013) or the change of C resources does not shift fungal community composition (Glassman et al. 2018).

Linking microbial diversity with functional genes coding for enzymes for decomposing different C compounds, we found that increasing bacterial diversity and fungal diversity enhance the potential decomposition of more complex C compounds (i.e. cellulose, hemicellulose, chitin, and lignin; Fig. 3). These results are to some extent consistent with our hypothesis that highly diverse microbial communities are more likely to obtain specialist species contributing to ‘narrow’ processes. An earlier study also found that the greater species richness contributes to a higher cellulose decomposition (Wohl, Arora and Gladstone 2004). The contribution of bacterial diversity on functional gene frequencies (Fig. 3) is also in line with a mesocosm study which shows that increasing bacterial richness leads to higher bacterial respiration (Bell et al. 2005). It is worth noting that the method for detecting functional genes was DNA-based, and thus the functional genes shown here only represent the potential functions, rather than the active microbial functions.

Given microbial diversity is often considered a ‘black box’ (Andrén and Balandreau 1999), the community structure inside the black box is obliged to be detected. Further analysis showed that a few bacterial taxa contribute to the functional genes coding for C degradation (Fig. 4A). Bacterial community composition is easily altered by the C substrates supplied, some of which are preferentially consumed by specific groups of bacteria (Cleveland et al. 2007). This is consistent with a study that demonstrated that bacterial community composition changes dramatically after being transferred to a new environment (with a different C supply) for 18 months (Glassman et al. 2018). Here our results revealed that most taxa of either bacteria or fungi failed to correlate with the potential genes (Fig. 4), indicating that many functions in different bacterial taxa are overlapping. For bacteria, merely Alphaproteobacteria, Gammaproteobacteria, and Chloroflexi contribute to the potential decomposition of hemicellulose and cellulose (Fig. 4A). Similarly, most taxa in fungal communities did not contribute to the potential functional composition (Fig. 4B). Compared with the most abundant classes Leotiomycetes and Agaricomycetes, the only ones that explained the potential decomposition of chitin, hemicellulose and cellulose were Taphrinomycetes, Umbelopsidomycetes, and Eurotiomycetes (Fig. 4B), which contributed to 2%-17% of the total abundance of the fungal community (data not shown). As hypothesized earlier, the decomposition of recalcitrant C compounds (narrow functions) is expected to be sensitive to shifts in community composition (Schimel et al. 1999). This once again indicated that the decomposition of recalcitrant substrates was determined by more specific taxa.

Our data were derived from a post-fire chronosequence where soil properties and physical environment evolved over time (Table 1). These physical and chemical factors explained the bulk of the variation in soil respiration. Key factors that explained the variations in respiration were SOM, DOC, and root biomass, which fed microorganisms and increased microbial diversity that subsequently influences CO_2_ emission (Fig. 1A). During the forest succession after a fire, the C supply increased along with the vegetation recovery, resulting in an increase in SOC contents, microbial biomass, and soil CO_2_ fluxes (Table 1 and Fig. 1). Microbial diversity was positively correlated with root biomass, indicating that the recovery of vegetation coverage brought along a higher diversity of microbes. This is more likely because the increase of root exudates will reshape the microbial community (Haichar et al. 2008, Huang et al. 2014).

Surprisingly, microbial biomass in the current study had no correlation with the CO_2_ emission (Fig. 1 and 2). This is probably because the decrease of microbial biomass was compensated by the increased soil temperature and drier conditions in the recently burned areas. To further test it, the multiple linear regression showed that microbial biomass significantly correlated with microbial respiration measured from the incubation experiment, while it has little contribution to soil respiration measured from the field (Table 2). As the incubation experiment was conducted at a specific condition for all areas, microbial respiration was inevitably determined by microbial biomass. In addition, microbial community composition will shift while incubating because of the changing environment. Species that adapt to the incubation condition will soon outcompete the ones that are not. However, in nature, microbial respiration can be affected by many factors, such as temperature, moisture, and the variety of microbial communities. Similar to our result that bacterial diversity significantly explained soil CO_2_ measured from the field (Table 2), previous studies also found that the taxonomic groups comprising the microbial biomass are more important than microbial biomass itself. For instance, Balser and Firestone (2005) found that microbial community composition of the soil was correlated with CO_2_ production, while not with microbial biomass. In addition, some studies found that the increase in microbial biomass hardly affects soil CO_2_ fluxes or C degradation (Glassman et al. 2018, Ge et al. 2019, Tan et al. 2021). It may also lie in the fact that heterotrophic respiration is not always directly proportional to microbial biomass when microbial density is saturated (Georgiou et al. 2017). In this case the respiration rate still increases with increasing C input, but no longer with microbial biomass (Georgiou et al. 2017). Despite our results showing microbial biomass had no correlation with CO_2_ emission, soil DOC concentrations were positively correlated with CO_2_ fluxes (Fig. 1).

In conclusion, by incorporating microbial diversity and the environmental variables, the predictive power of models on soil C emission is significantly improved. Changes in bacterial Shannon diversity explain more variation of soil C emissions than that in fungal Shannon diversity, which may indicate that fungi species are more multifunctional than bacteria. Furthermore, potential decompositions of recalcitrant C, notably hemicellulose, cellulose, and chitin, were altered by the increasing microbial diversity, but not with microbial taxa groups. Here, the study was applied in summer which reflects the highest microbial activity and C input of the studied areas, indicating the contributions of microbial diversity and DOC on soil repiration in other seasons may be less strong. In addition, we note that our results were obtained in boreal forests after fire disturbances, therefore, some paths in the SEM model may not apply to or may be found to be stronger in other ecosystems. Nevertheless, we reckon that the contribution of microbial diversity, notably bacterial diversity, to soil C decomposition reflects a general pattern in soil ecosystems. This study predicts the response of ecosystems to the changes in microbial diversity and the environment and understands how fire disturbances affect microbial diversity and decomposition, and in turn, regulate soil biogeochemical processes.

## Supplementary Material

fiac074_Supplemental_File
