# Supplementary material for: Microbial biodiversity contributes to soil carbon release: a case study on fire disturbed boreal forests
Source: FEMS Microbiol Ecol. 2022 Jun 24;98(8):fiac074. doi: 10.1093/femsec/fiac074 (PMC9303362; doi:10.1093/femsec/fiac074)
Supplement: fiac074_Supplemental_File [file fiac074_supplemental_file.docx]

Figure S1. ﻿Relative abundance (%) of bacterial phyla (A; average abundance of each area and depth; n = 9) and of fungal classes (B) across four forest areas of two soil layers.

Figure S2. ﻿Results of the non-metric multidimensional scaling (NMDS) of the

sequencing data of bacteria (A) and fungi (B). Distance matrices were based on the Bray-Curtis distances shows the clustered bacterial and fungal communities at the OTU level. Red circles represent samples from Fire_3_, green represent Fire_25_, blue represent Fire_46_ and purple represent Fire_100_. The circular points represent samples of the 5 cm layer (organic soils), and the triangular points represent samples from the 30 cm layer (mineral soils).

Figure S3. Bacterial Shannon index calculated using (A) original OTUs and (B) the top 95% abundant OTUs, and fungal Shannon index calculated using (C) original OTUs and (D) the top 95% abundant OTUs.

Table S1. Data that were log-transformed to correspond with normality.

| Variables | Data transformation | Normality condition |
| --- | --- | --- |
| Temp (oC) | No | Yes |
| Activ.layer.depth (m) | No | Yes |
| AvMoi (%) | ln(*x*+0.001) | Yes |
| SOM (g g^-1^) | No | Yes |
| Roots (g g^-1^) | ln(*x*+0.001) | Yes |
| DOC (mg g^-1^) | ln(*x*+0.001) | Yes |
| MB (mg g^-1^) | No | Yes |
| H_Bact_ | No | Yes |
| H_Fungi_ | No | Yes |
